# A predictive model for recurrence after upfront surgery in patients with resectable pancreatic ductal adenocarcinoma (PDAC) by using preoperative clinical data and CT characteristics

**DOI:** 10.1186/s12880-022-00823-4

**Published:** 2022-07-03

**Authors:** Ningzi Tian, Dong Wu, Lei Zhu, Mengsu Zeng, Jianke Li, Xiaolin Wang

**Affiliations:** 1 Shanghai Institute of Medical Imaging, No. 180 Fenglin Road, Xuhui District, Shanghai, 200032 China; 2 Department of Radiology, Zhongshan Hospital, Fudan University, No. 180 Fenglin Road, Xuhui District, Shanghai, 200032 China

**Keywords:** Pancreatic ductal adenocarcinoma, Tomography computed, Nomogram

## Abstract

**Background:**

The overall survival for patients with resectable PDAC following curative surgical resection hasn’t been improved significantly, as a considerable proportion of patients develop recurrence within a year. The purpose of this study was to develop and validate a predictive model to assess recurrence risk in patients with PDAC after upfront surgery by using preoperative clinical data and CT characteristics.

**Methods:**

The predictive model was developed based on a retrospective set of 141 pancreatic cancer patients after surgery. A separate set of 77 patients was used to validate model. Between January 2017 and December 2019, all patients underwent multidetector pancreatic CT and upfront surgery. Univariable and multivariate Cox regression was used to determine the risk factors related to recurrence and then establish a nomogram to estimate the 1-year recurrence probability. The Harrell C-index was employed in evaluating the discrimination and calibration of the model.

**Results:**

A total of 218 patients in this retrospective cohort. A recurrence model in nomogram form was developed with predictors including tumor size (hazard ratio [HR], 1.277; 95% CI 1.098, 1.495; *P* = 0.002), tumor density in the portal vein phase (HR, 0.598; 95% CI 0.424, 0.844; *P* = 0.003), peripancreatic infiltration (HR, 4.151; 95% CI 2.077, 8.298; *P* < 0.001), suspicious metastatic lymph node (HR, 2.561; 95% CI 1.653, 3.967; *P* < 0.001), Neutrophils/Lymphocytes ratio (HR, 1.111; 95% CI 1.016, 1.215; *P* = 0.020). The predictive nomogram had good discrimination capability with these predictors with an area under curve at 1 year of 0.84 (95%CI 0.77, 0.91) in the development set and 0.82 (95% CI 0.72, 0.92) and 0.84 (95% CI 0.74, 0.94) in the validation set for two radiologists reading respectively.

**Conclusions:**

The model developed based on preoperative clinical data and CT characteristics of resectable pancreatic ductal adenocarcinoma patients, which can helpfully estimate the recurrence-free survival. It may be a useful tool for clinician to select optimal candidates for upfront surgery or neoadjuvant therapy.

## Introduction

Pancreatic cancer is the fourth leading cause of cancer death worldwide, and its incidence has been increasing over the years [1, 2]. The 5-year survival rate is less than 6% [3]. In patients with pancreatic ductal adenocarcinoma (PDAC), curative surgical resection is still the primary therapy option for long-term survival. However, the prognosis for patients following curative surgical resection hasn’t been improved notably, with a considerable proportion of patients experiencing locoregional and/or distant recurrence within a year [4–6]. A shorter time (< 1 year) to recurrence after resection was significantly associated with poor overall survival [5, 7–10].

As for resectable PDAC, the standard treatment option is upfront surgery, which means performing curative surgery before chemotherapy or radiation therapy, and then followed by adjuvant chemotherapy [11]. Recently, neoadjuvant therapy also has been recommended for high-risk resectable PDAC patients by National Comprehensive Cancer Network (NCCN) guidelines [12]. However, the criteria for patients at high risk remains unclear. Therefore, biomarkers that can be measured easily and reliably are essential for evaluation of tumor aggressiveness and improvement in the selection of patients with high-risk probabilities for recurrence when making treatment strategies for resectable PDAC.

Contrast-enhanced pancreatic CT is the primary option to assess the tumor staging and resectability before treatment [13]. Moreover, the pancreatic tumor characteristics of CT images, clinical data and laboratory parameters have potential prognostic value for patients with resectable PDAC [14]. Previous research referenced predictors focused on postsurgical factor such as tumor size, degree of differentiation, surgical margin and so on, but some of which couldn’t be known when making treatment strategy initially.

This study aimed to assess whether CT conventional characteristics of the tumor could be evaluated preoperatively associated with early recurrence for patients with resectable PDAC. A predictive model for ****established based on these parameters was essential for treatment decision.

## Materials and methods

### Patient’s selection

This retrospective study was approved by our hospital Ethics Committee and the need for informed patient consent was waived. We used TRIPOD (transparent reporting of a multivariable prediction model for individual prognosis or diagnosis) guidelines to guaranteeing the rigor and standard of this study [15].

**Fig. 1:**
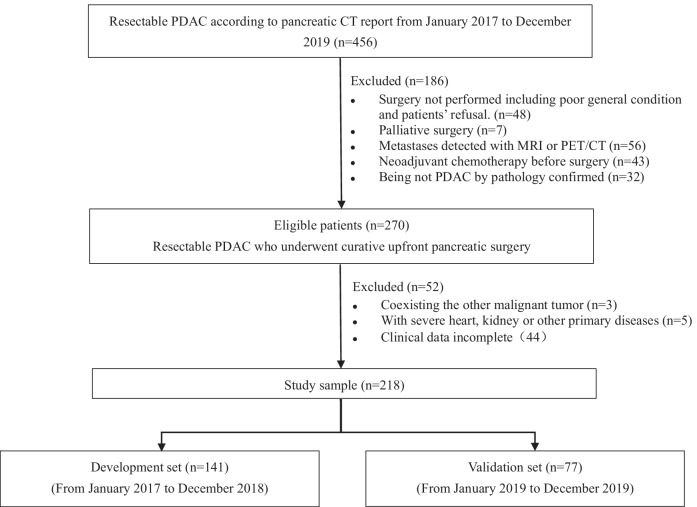
Study flow chart for development and validation set. After exclusion, 218 of 454 patients were identified in our study

This study flow chart was shown in Fig. 1, descripted the patient selection process and exclusion criterion. We reviewed the contrast-enhanced pancreatic CT reports in hospital database from January 2017 to December 2019 and consecutively registered the patients with resectable PDAC. According to the NCCN criteria, the criteria of pancreatic cancer resectability is the tumor with no contact celiac artery, superior mesenteric artery, and common hepatic artery, and no contact or ≤ 180° contact with the portal vein or superior mesenteric vein without vein contour irregularity. Each radiologic and medical record was reviewed by two experienced radiologists (W.D. and Z.L., with 20 and 8 years of working experience in radiology department respectively). The exclusion criteria were as follows: (a) surgery not performed or underwent palliative surgery; (b) metastases detected with other imaging scan; (c) being not PDAC by pathology confirm; (d) coexisting other malignant tumor or severe other primary diseases. Cases with incomplete clinical data also were excluded. In the final cross-sectional study sample, 218 eligible patients were enrolled. They were divided into the development set and the validation set according to the time of performing surgery.

### Clinical variables

Based on previous reported, demographic and clinical variables were chosen as potential variables associated with prognosis of patients with pancreatic cancer. Preoperative laboratory variables comprised carbohydrate antigen 19-9 (CA19-9), total bilirubin, lymphocytes, neutrophils, platelets, and C-reactive protein were routinely measured within one week before surgery. About pathology findings, we recorded the following information from surgical specimens: type of pancreatic surgery, grade of differentiation, lymphvascular invasion, perineural invasion. Pathologic tumor stage was complied with the eighth edition of the American Joint Committee on Cancer (AJCC) staging system [16].

### CT scans and image variables

Pancreatic cancer NCCN guidelines suggested that CT scan should contain unenhanced imaging, arterial phase (AP) and portal venous phase (PVP) imaging [12]. The Contrast-enhanced CT scan were performed by using the Aquilion ONE CT (Toshiba Medical Systems Corporation) and Light Speed VCT (GE Healthcare). The scanning parameters were as follows: 0.5–3 mm slice thickness; in-plane resolution of 0.5 $\times$ 0.5 mm or 0.625 $\times$ 0.625 mm/pixel; data reconstructed at 2–5 mm intervals; tube voltage 100–120 kV and tube current 100–150 mA. Images were obtained after intravenous administration of 80–100 ml of 300 mg of iodine per milliliter of nonionic contrast material (Ultravist 300; Schering) using a power injector through an 18-gauge at a rate of 3–5 ml/s. The arterial phase and portal vein phase were started at 20–35 s and 60–75 s, respectively, after injection.

CT variables were chosen from PDAC radiology reporting template, which included tumor location, size, enhancement pattern, tumor necrosis, peripancreatic infiltration, tumor contact with major vessel, adjacent organ invasion, and suspicious metastatic lymph node [17]. The tumor density in the AP and PVP was recorded as hypo-, iso-, or hyperdense, which was evaluated compared to the pancreatic parenchyma. Tumor tissue without enhance after intravenous contrast material administration was considered as necrosis area. All image variables were assessed by two radiologists (W.D. and Z.L.). They didn’t know postsurgical pathologic findings and outcome data.

### Outcome measures

Recurrence-free survival was defined as the date from curative surgical resection to recurrence, metastasis or death. Tumor recurrence was defined as newly detected locoregional and/or distant metastatic tumors based on the findings of CT, MRI, PET/CT or US with or without increased serum carcinoembryonic antigen (CEA), carbohydrate antigen 125(CA125) or CA19-9 levels. Curative surgical resection was performed by specialized surgeon (50-arounded pancreatic cancer surgeries annually). All patients were followed up with biochemical and imaging examination assessment every 3 to 6 months until March 2021.

### Statistical analysis

All statistical analyses were performed with SPSS software (version 20.0) and RStudio (version 4.0.4). Patient characteristics of this study sample were described by means and standard deviations for continuous variables and frequencies and percentages for categorical variables. The consistency evaluation between the two sets was applied with Fisher exact, *t* test, χ^2^ validation, validation, or analysis of variance according to data type. Multivariable Cox proportional hazards models to filter risk factors association with RFS. The hazard ratio (HR) and corresponding 95% confidence intervals were estimated. *P* < 0.05 was considered to indicate statistical significance. Interobserver agreement was quantified using the kappa statistic for categorical variables of CT characteristic. The kappa value of more than 0.6 was considered reliable.

In the development set, all covariates in Cox model were selected as risk factor by stepwise regression. The nomogram was generated with the independent risk factors for predicting the 1-year RFS. The probabilities of recurrence were read according to the nomography. Time-dependent receiver operating characteristic curve was plotted to assess the discrimination of our model in development and validation sets respectively.

## Results

### Patient characteristics

All 456 patients with PDAC were screened and 270 patients satisfied with eligibility. 52 patients were excluded for coexisting other malignant tumor, severe primary diseases, and clinical data incomplete. Finally, the study sample comprised 218 patients and was divided into the development set (n = 171; mean age, 63.43 ± 9.25 years; 87 male) and the validation set (n = 77; mean age, 64.49 ± 10.40 years; 41 male). The patient characteristics are summarized as following (Table 1). As shown in Tables 1, the distribution of laboratory results such as is similar in the two sets (*P* > 0.05). Because of cross-sectional study, it’s can be explained that follow-up duration in the development set was longer than the validation set (median, 33 months [rang, 2–48 months] vs median, 18 months[rang, 6–24 months]; *P* < 0.001). Tumors recurred in 96 of 141 (68.1%) patients in the development set and 44 of 77 (57.1%) patients in the validation set during follow-up period. The median RFS was 17.84 months (ranging from 1.08 to 48.03 months) in the development set and 14.23 months (ranging from 2.37 to 24.48 months) in validation set. Adjuvant therapy were initiated 4–8 weeks after surgery depending on their general condition in 165 patients. The adjuvant therapy regimens after surgery included gemcitabine (15/165), 5-FU/leucovorin (6/165), gemcitabine + capecitabine(10/165), S-1(70/165) and gemcitabine + albumin-bound paclitaxel(64/165). Adjuvant therapy performed was same frequent in the development and validation sets (75.18% [106 of 141] vs 76.62% [59 of 77]; *P* = 0.812).

**Table 1:** Patients’ characteristics of the study sample

Characteristic	Development Set (n = 141)	Validation Set (n = 77)	*P* Value
Age (years)^a^	63.43 ± 9.25	64.49 ± 10.40	0.277^#^
*Gender*			0.226
Male Female	87(61.70) 54(38.30)	41(53.25) 36(46.75)	
*Tumor* *location*			0.200
Head	88(62.41)	40(51.95)	
Body	19(13.48)	17(22.08)	
Tail	34(24.11)	20(25.97)	
Laboratory results^*^			
Cancer antigen 19–9(U/ml)	141.2(0.6–7767)	92.1(2–10,000)	0.573
Bilirubin (μmol/L)	13.8(3.7–436.1)	13.2(3.8–450.4)	0.334
Neutrophils/Lymphocytes	2.49 (0.52–13.25)	2.41(0.91–4.99)	0.479
Platelets/Lymphocytes ratio	134.12(61.07–463.33)	137.06(2.4–428)	0.985
C-reactive protein(mg/L)	1.2(0.3–32.9)	1.6(0.3–381.2)	0.294
Adjuvant therapy performed^Σ^	106(75.18)	59(76.62)	0.812
*Type of pancreatic surgery*^£^			0.924
Standard pancreaticoduodenectomy	54 (38.30)	29 (37.66)	
Standard distal pancreatectomy	52 (36.88)	28 (36.36)	
Extended pancreaticoduodenectomy	26 (18.44)	14 (18.18)	
Extended distal pancreatectomy	8 (5.67)	6(7.79)	
Standard total pancreatectomy	1 (0.71)	0(0.00)	
Negative resection margin (R0)	140(99.29)	76()	0.663
*Primary* *tumor* *(T)* *stage*			0.900
T1	44 (31.21)	22(28.57)	
T2	79 (56.03)	44(57.14)	
T3	18(12.76)	11(14.29)	
*Regional lymph node (N) stage*			0.064
N0	80 (56.74)	54(70.13)	
N1	53 (37.59)	17(22.08)	
N2	8 (5.67)	6(7.79)	
*AJCC prognostic stage group*			0.090
IA	29(20.57)	12(15.58)	
IB	41(29.08)	32(41.56)	
IIA	10(7.09)	8(10.39)	
IIB	51(36.17)	17(22.08)	
III	9(6.38)	5(6.49)	
IV	1(0.71)	3(3.90)	
*Tumor differentiation*			0.108
Well differentiated	0(0.00)	0(0.00)	
Moderately differentiated	70(49.65)	47(61.04)	
Poorly or undifferentiated	71(50.35)	30(38.96)	
Lymphovascular or microvascular invasion present	27(19.15)	18(23.38)	0.461

Data are numbers, with percentages in parentheses, unless otherwise specified

^a^Data are means ± standard deviations

*Data are medians, with ranges in parentheses

^Σ^53 patients didn’t receive adjuvant therapy because of underlying diseases and perioperative complications

^£^Type of surgery was classified as standard surgery or extended surgery involving concomitant vein or additional organ resection

^#^Fisher’s exact test, χ^2^ test or one-way analysis of variance were used to compared data, excepted where indicated

### Univariate and multiple Cox regression analysis

Through Cox proportional hazard analysis, independent risk factors were selected associated with recurrence including: tumor size (hazard ratio [HR], 1.277; 95% CI 1.098, 1.495; *P* = 0.002), tumor density in the portal vein phase (HR, 0.598; 95% CI 0.424, 0.844; *P* = 0.003), peripancreatic infiltration (HR, 4.151; 95% CI 2.077, 8.298; *P* < 0.001), suspicious metastatic lymph node (HR, 2.561; 95% CI 1.653, 3.967; *P* < 0.001), Neutrophils/Lymphocytes ratio (HR, 1.111; 95% CI 1.016, 1.215; *P* = 0.020) (Table 2).

**Table 2:** Using Cox proportional hazard analyses for postsurgical RFS in development set

Parameter	Univariable cox proportional hazard analysis			Multivariable cox proportional hazard analysis		
	Regression coefficient	Hazard ratio	*P* value	Regression coefficient	Hazard ratio	*P* value
Age	− 0.016	0.984(0.963,1.006)	0.157			
Male sex	0.063	1.065(0.705,1.608)	0.766			
Tumor size(cm)	0.349	1.417(1.222,1.644)	< 0.001	− 0.248	1.277(1.098,1.495)	0.002
Dominant location			0.165			
Head	− 0.105	0.901(0.566,1.434)	0.659			
Body	− 0.721	0.486(0.227,1.042)	0.486			
Tail	1	1[reference]	–			
Tumor density in AP			0.747			
Hyperdense	1	1[reference]	–			
Isodensel	0.301	1.351(0.407,4.491)	0.623			
Hypodense	0.200	1.020(0.373,2.787)	0.969			
Tumor density in PVP			< 0.001	− 0.940	0.598(0.424,0.844)	0.003
Hyperdense	1	1[reference]	–			
Isodensel	0.407	1.503(0.714,3.163)	0.283			
Hypodense	1.141	3.129(1.526,6.416)	0.002			
Tumor necrosis (Yes/No)	0.104	1.110(0.451,2.733)	0.821			
Peripancreatic infiltration (Yes/No)	− 1.489	0.226(0.116,0.438)	< 0.001	1.423	4.151(2.077,8.298)	< 0.001
Contact to SMV or PV	− 0.228	0.431(0.451,1.405)	0.431			
Suspicious metastatic lymph node	− 1.089	0.336(0.223,0.507)	< 0.001	0.940	2.561(1.653,3.967)	< 0.001
Cancer antigen 19–9	0.000	1(1,1)	0.009	0.000	1(1,1)	0.430
Bilirubin	0.003	1.003(1.001,1.005)	0.011			
Neutrophils/Lymphocytes ratio	0.138	1.148(1.066,1.237)	< 0.001	0.105	1.111(1.016,1.215)	0.020
Platelets/Lymphocytes ratio	0.000	1(1,1)	0.835			
C-reactive protein(mg/L)	− 0.042	0.959(0.905,1.016)	0.159			

Data in parentheses are 95% confidence intervals

*AP* Arterial phase, *PV* portal vein, *PVP* portal venous phase, *SMV* superior mesenteric vein

### Nomogram

**Fig. 2:**
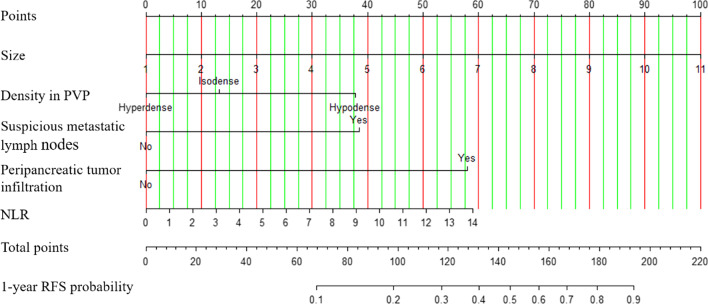
The nomogram for predicting 1-year recurrence probability of PDAC patients with upfront surgery

**Fig. 3:**
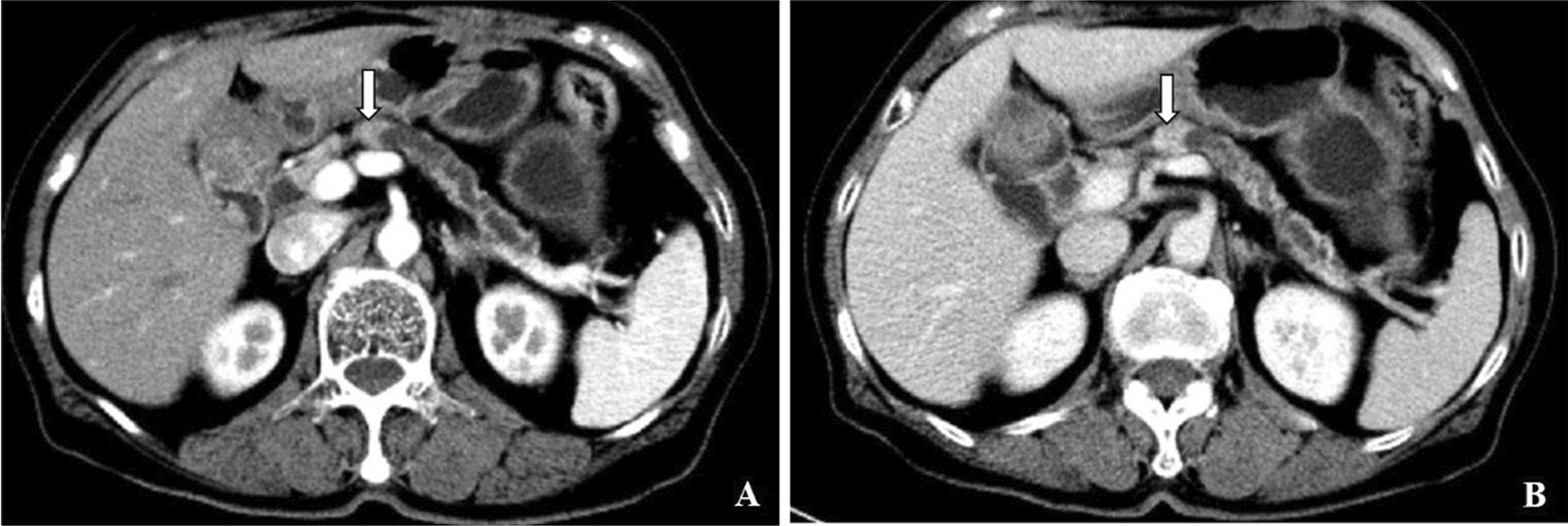
A 73-year-old woman with no discomfort and was admitted to our hospital for pancreatic mass detected by CT examination. Contrast-enhanced CT images show a mass in pancreatic head (arrow) with diameter 1.4-cm, hyperdense in **A** arterial phase and **B** portal venous phase. Tumor infiltration and enlarged lymph nodes are absent. NLR is 1.12. Patient was alive for 40 months until our last follow-up after standard pancreaticoduodenectomy with no tumor recurrence

**Fig. 4:**
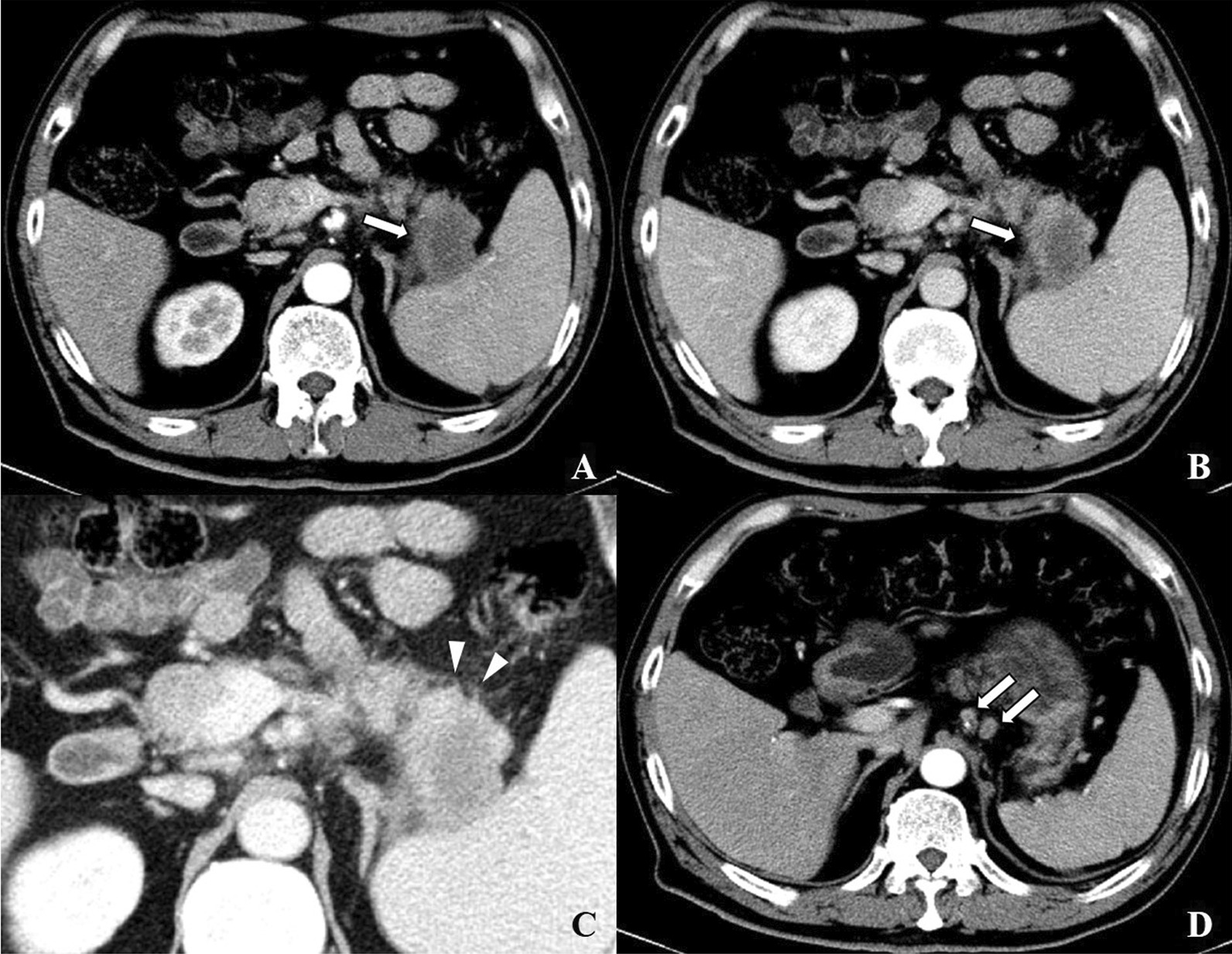
A 62-year-old man with elevated CA19-9. Preoperative CT detected a solid lesion in pancreatic tail. Moderately differentiated pancreatic ductal adenocarcinoma was confirmed after extended distal pancreatectomy. Contrast-enhanced CT images demonstrate a 4.6-cm hypodense mass in pancreatic tail (arrow) (**C**) in (**A**) arterial phase and (**B**) portal venous phase. Peripancreatic infiltration is appeared(arrowhead). Two enhanced suspicious metastatic lymph nodes (**D**) are observed (arrow). NLR is 2.96. Tumor recurrence occurrence 5.6 months and died 15.1 months after surgery

The nomogram was established based on tumor size, tumor density in PVP, suspicious metastatic lymph nodes, peripancreatic tumor infiltration and NLR (Fig. 2). In the development set, discrimination capability of model with the AUC of 0.84(95% CI 0.77, 0.91) is good and the calibration slope is 0.99. The probability of 1-year recurrence can be read from the nomogram directly. For example, a woman is with resectable PDAC. A diameter 1.4 cm mass (5 points) in pancreatic head appearing hyperdense (0 points) in PVP. There is no suspicious metastatic lymph node (0 points), no peripancreatic tumor infiltration (0 points) and NLR of 1.12 (5 points). The total nomogram points are 10 and a very low (< 0.1) probability of 1-year recurrence (Fig. 3). For another case, a resectable PDAC patients with a 4.6 cm(35 points)mass, hypodense in PVP (38 points), suspicious metastatic lymph node (38.5 points), peripancreatic tumor infiltration (58 points) and NLR of 2.96 (13 points) would have a total points of 182.5 and a 0.8 probability of 1-year recurrence (Fig. 4).

### Nomogram performance assessment

**Fig. 5:**
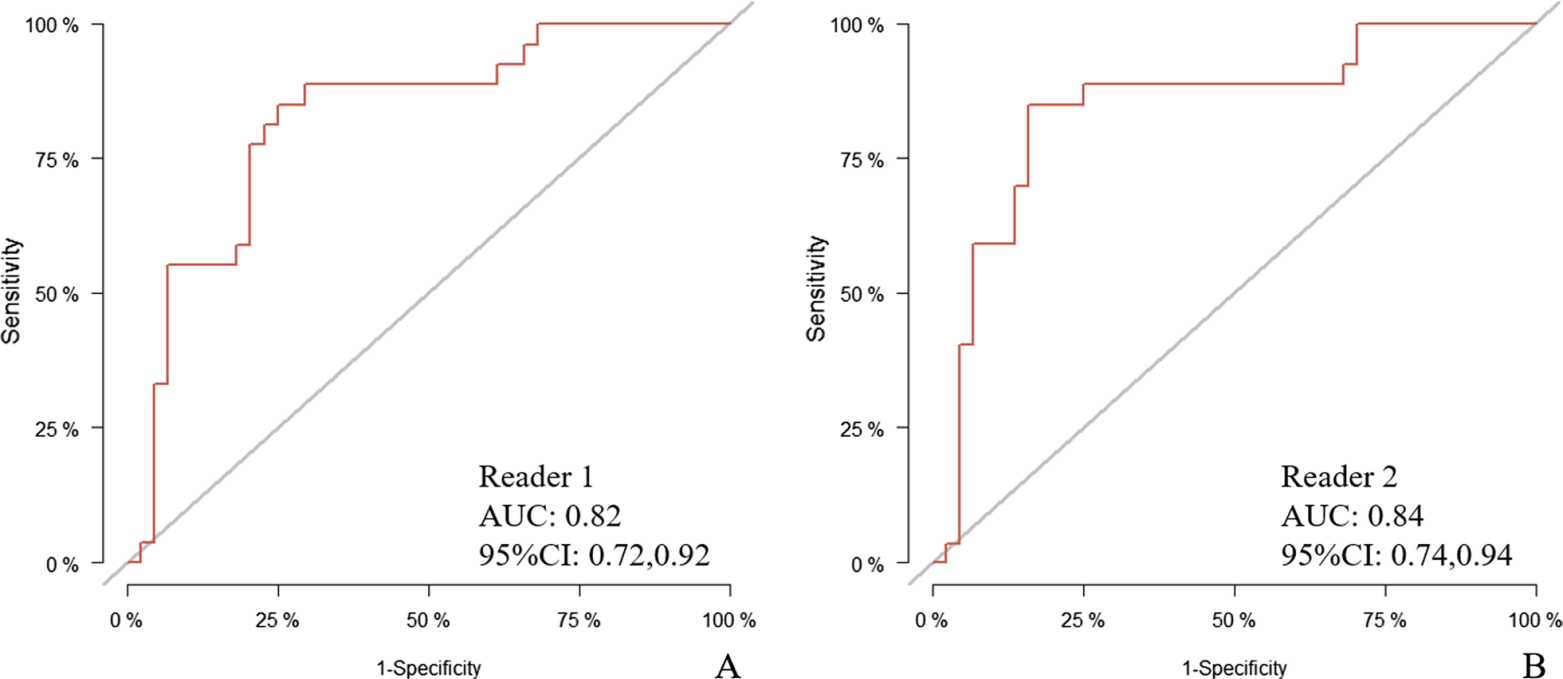
The AUC was used to interpret the model performance in validation set of reader 1 (**A**) and reader 2 (**B**)

The risk nomogram points reliably predicted discrimination capability with the area under curve (AUC) for predicting 1-year RFS probability of 0.82(95% CI 0.72, 0.92) for one reader and 0.84 (95% CI 0.74, 0.94) for another reader in validation set. Calibration curves showed the agreement between predicted and observed probabilities of 1-year RFS after upfront surgery in both sets (Fig. 5).

## Discussion

In the present study, we developed and validated a predictive model in patients with resectable PDAC, based on the routinely measured clinical factors and tumor characteristic at CT images available within two weeks before the surgery. The performance of this model was satisfactory in discrimination aspects in both the development and validation sets. Previous predictive models had limited clinical utility as depending on postsurgical pathology findings to some extent [10, 18, 19]. Moreover, some researchers investigated the association between PDAC imaging features and clinical outcomes by quantitative or qualitative methods [20–23]. In clinical practice, quantitative measurement and radiomics labels are not widely used. In contrast, our model is based on qualitative features, data easily available and have high clinical practicability.

As non-invasive imaging assessment, now contrast-enhanced pancreatic CT scans play an important part when we decide treatment regimen for patients with pancreatic cancer. According to the pancreatic CT preoperatively, we observed a significant association between factors of the tumor size, tumor density in the portal venous phase, suspicious metastatic lymph node, peripancreatic infiltration and NLR and the RFS. These imaging and clinical factors indicated the tumor development and progression of PDAC. Previous studies have provided direct and indirect evidence of potential correlations of morphologic characteristics of pancreatic cancer on CT with patients’ outcome [22, 24]. Stromal of the pancreas associated with biological characteristics can be observed on CT images. The enhancement patterns of PDAC tumors are related to their dense desmoplastic stromal reaction of the pancreas [22, 25]. This finding that hypodensity in PVP indicate the short RFS again suggests a role of the stroma in tumor progression and metastasis, consistent with other literature [26]. The five factors we used in the model are easily acquired in clinical datasets. So, this model can be provided as an accessible tool for clinicians to assess patients’ risk of recurrence. Based on the risk of recurrence within a year, patients with resectable PDAC might be suggested to perform upfront surgery or neoadjuvant therapy initially [27].

Preoperative NLR as an only clinical factor is in our model. Some studies have identified that inflammation is participated in outcome in patients with cancer. The neutrophils play important roles in systemic inflammatory response, which promote tumor growth, facilitate tumorigenesis, metastasis and stimulate tumor angiogenesis [28, 29]. Stotz et al. found that advanced tumor stage and high NLR (> 5) were independent prognostic marks for operable pancreatic tumors in their research, which used a multivariate Cox proportional-hazard model [30]. Multiple studies have investigated that the NLR was a predictive marker in survival prognosis of pancreatic invasive carcinoma [29, 31–33]. This relationship could explain the correlation between high NLR and short RFS in our current study.

CA19-9, CEA and CA125 are commonly considered as tumor biomarkers for the prognosis of pancreatic cancer, among which CA19-9 is the most valuable factor used for auxiliary diagnosis and recurrence monitoring and correlated with clinical course of disease [34–36]. However, in our predictive model, the CA19-9 was absent. When we tried to include this parameter to the final predictive model with other parameters. The accuracy of the new model was not improved compared to the current model. At last, we excluded it from the predictive model after serious consideration. One reason may be that CA19-9 level can also elevated in some patients with biliary infection, inflammation, and obstruction, which confound the survival outcome.

Several limitations in this study should be acknowledged. First, retrospective single-institution study was more prone to bias than prospective study, despite our efforts to minimize selection bias and avoid bias from missing data. Second, during the follow-up period, the consistency in determining recurrence in each patient is absent. Only a few patients had recurrence masses that were confirmed by pathologic finding. In many other cases, it’s according to symptoms, the increasing of tumor biomarkers or radiologic findings to diagnose disease relapse. In addition, a longer follow-up period is needed and enrolled patients rechecked in scheduled visits should be ensured. Third, in our predictive model, a critical point for distinguish between low-risk and high-risk groups need to furtherly determined.

## Conclusions

The model developed mainly based on preoperative clinical data and CT characteristics of resectable pancreatic ductal adenocarcinoma patients can helpfully estimate the RFS, which may be a useful tool for clinician to select patients for upfront surgery or neoadjuvant therapy.

## Data Availability

Due to patient privacy protection, materials and data are not publicly available but are available from the corresponding author for reasonable request.

## Abbreviations

PDAC: Pancreatic ductal adenocarcinoma
CT: Computed tomography
RFS: Recurrence-free survival
MRI: Magnetic resonance imaging
PET: Positron emission tomography
AFP: Alpha-fetoprotein
CA19-9: Cancer antigen 19–9
CEA: Carcinoembryonic antigen
CA125: Carbohydrate antigen 125
NLR: Neutrophils/Lymphocytes ratio
AP: Arterial phase
PVP: Portal venous phase
